# Liver Abscesses in Patients With Beta Thalassaemia Major: A Case Series and Mini-Review of the Literature

**DOI:** 10.7759/cureus.82258

**Published:** 2025-04-14

**Authors:** Nilanga Nishad, Madunil A Niriella, Arjuna P de Silva, Hiruni Jayasena, Vajira T Samarawickrama, Kunchana Thebuwana, Dhananja Namalie, Harsha Perera, Anuja P Premawardhena, Hithanadura J de Silva

**Affiliations:** 1 Department of Gastroenterology and Hepatology, University Medical Unit, Colombo North Teaching Hospital, Faculty of Medicine, University of Kelaniya, Ragama, LKA; 2 Department of Gastroenterology and Hepatology, Sheffield Teaching Hospitals NHS Foundation Trust, Sheffield, GBR; 3 Department of Medicine, University Medical Unit, Colombo North Teaching Hospital, Faculty of Medicine, University of Kelaniya, Ragama, LKA; 4 Department of Hepatology and Liver Transplant, Colombo North Centre for Liver Diseases, Ragama, LKA; 5 Department of Medicine, General Sir John Kotelawala Defence University, Ratmalana, LKA; 6 Department of Gastroenterology and Hepatology, East Surrey Hospital, Surrey and Sussex Healthcare NHS Trust, Redhill, GBR; 7 Department of Medical Microbiology, Colombo North Teaching Hospital, Ragama, LKA; 8 Department of Medical Microbiology, Public Health Wales Microbiology Division, Glangwili Hospital - Hywel Dda University Health Board, Carmarthen, GBR; 9 Department of Clinical Medicine, Faculty of Medicine, University of Kelaniya, Ragama, LKA; 10 Department of Medicine, Hemals Adolescent and Adult Thalassaemia Care Center, Kadawatha, LKA; 11 Department of Internal Medicine, Faculty of Medicine, University of Kelaniya, Ragama, LKA

**Keywords:** antibiotic resistance (abr), antibiotics therapy, covid 19, liver abscess, thalassaemia

## Abstract

Treatment of liver abscesses in patients with transfusion-dependent thalassaemia remains a challenging task due to the interplay of multiple factors, including difficult venous access and the co-existence of other organ dysfunction, such as diabetes mellitus. We report case histories of three transfusion-dependent thalassaemia patients with liver abscesses, two of whom had repeated episodes of the disease. We recommend a prolonged regimen of intravenous and oral antibiotics to eliminate the infection, along with more vigilant and regular follow-up using imaging for early detection of recurrence. Finally, we highlight the importance of maintaining continuous venous access - another often overlooked challenge among thalassaemia patients.

## Introduction

Beta thalassaemia is the most common single-gene disorder globally. Unsurprisingly, it has become the most clinically significant monogenic disorder in the Sri Lankan context, with approximately 2,500 patients seeking treatment and over 600,000 heterozygotes. Cardiac disease due to iron overload remains the leading cause of death among those with thalassaemia major, while severe infections have risen in prominence as a cause of both morbidity and mortality worldwide [1-5].

Multiple factors increase the vulnerability to infections in thalassaemia patients. Iron overload is thought to increase the likelihood of bacterial sepsis, while increased iron favours the growth of siderophilic bacteria such as *Yersinia*, *Klebsiella* spp., *Salmonella typhi*, and *Escherichia coli*. Splenectomy, which is not uncommonly carried out in patients with severe thalassaemia, increases susceptibility to infections caused by encapsulated bacteria severalfold. Frequent blood transfusions predispose patients to transfusion-transmitted infections. Diabetes mellitus, which is a sequela of iron overload, further increases the risk of infections. The incidence of liver abscesses in the general population is 23 per million, with male gender and increasing age suggested as risk factors, although abscesses can occur without an identifiable cause [6,7]. No studies have estimated the prevalence of liver abscesses in patients with thalassaemia major.

Liver abscess is a serious infection, and its treatment is always a challenging task for physicians. A liver abscess in a thalassaemia patient presents an even greater management challenge. Unfortunately, no specific guidelines related to the treatment of liver abscesses in thalassaemia have been published. We present three patients with transfusion-dependent thalassaemia who developed liver abscesses and review the literature on the topic, highlighting the complexities associated with its treatment.

This article was published on the preprint server, Research Square, on December 28, 2022 (10.21203/rs.3.rs-2292650/v1).

## Case presentation

Case 1

A 30-year-old male β-thalassaemia major patient, who had been on monthly blood transfusions since the age of five, presented with a history of right upper abdominal pain and high fever with chills lasting over a week. He had diabetes mellitus with poor glycaemic control and had undergone splenectomy at the age of 19 years. Iron overload-related cirrhosis of the liver had been detected at age 25. Hepatitis B virus (HBV) and hepatitis C virus (HCV) antibodies were negative. Although he was up to date on post-splenectomy vaccinations, over the past 10 years he had required hospital admissions for two severe infections - one for a lower respiratory tract infection and another for cellulitis of the lower limb [8].

At presentation, his haemoglobin level was 5.5 g/dL, white blood cell (WBC) count was 22 × 10³, platelets were 319 × 10³, and C-reactive protein was 32 mg/dL. Liver function, coagulation, and renal tests were normal, except for serum albumin of 2.5 g/dL and total bilirubin of 60 µmol/L (direct bilirubin of 24 µmol/L). The last recorded serum ferritin, prior to this illness, was 2,490 ng/mL. Retroviral and melioidosis antibodies were negative, and the Mantoux test was non-reactive.

**Figure 1:**
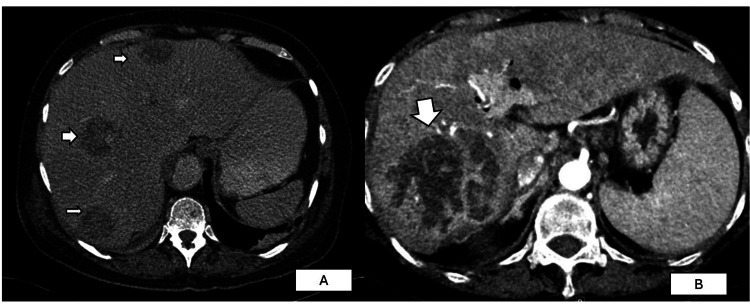
Computer tomography images of the pyogenic liver abscess (A) Patient 1: Arrowheads show liver abscesses in Segments 4a, 7, and 8; (B) Patient 2: An arrowhead shows a large abscess in Segments 6/7.

An abdominal ultrasound scan (USS) revealed two hypoechogenic lesions in Segments 6 and 8 of the liver, suggestive of liver abscesses. Subsequent contrast-enhanced computed tomography (CECT) showed six hypoattenuating focal liver lesions in Segments 4a, 5, 6, 7, and 8, with the largest measuring 2.2 × 2 cm in Segment 8 (Figure 1). A small calculus was detected within the cystic duct without radiological evidence of cholecystitis. Blood cultures and abscess aspirate samples grew *Klebsiella pneumoniae* after eight days.

He was treated with intravenous meropenem 1 g three times daily for 19 days and intravenous ciprofloxacin 400 mg twice daily for 22 days. As he improved clinically and biochemically, with the normalization of inflammatory markers, the patient was discharged on oral ciprofloxacin 500 mg twice daily for two weeks. Ten months later, he developed similar symptoms. His WBC count was 31.5 × 10³, platelets were 318 × 10³, haemoglobin was 8.3 g/dL, and C-reactive protein was 62 mg/dL. Repeat USS of the abdomen revealed two septated cystic lesions measuring 1.7 × 2.2 cm and 1.7 × 2.1 cm, respectively. USS-guided aspiration was deemed impractical by the specialist radiologist, and a decision was made to treat empirically with broad-spectrum antibiotics - intravenous imipenem 1 g three times daily and intravenous metronidazole 500 mg three times daily - while awaiting the blood culture report. However, the patient self-discharged before completing the intravenous antibiotics. He was re-admitted three days later with a high swinging fever, markedly elevated blood sugar levels, and dyspnoea at rest, with an oxygen saturation of 88% on face mask oxygenation. Further investigations confirmed COVID-19 pneumonia in addition to liver abscesses (abdominal scan findings were similar to the previous admission, and repeat blood cultures again grew *K. pneumoniae* after 15 days). The same antibiotic regimen was re-initiated after repeating blood cultures. Unfortunately, he succumbed to the illness in an ICU setting two days later (Table 1).

**Table 1:** Comparison of the patient characteristics

Age and Gender	30-Year-Old Male	26-Year-Old Male	46-Year-Old Male	Reference Range (Male/Female)
Underlying Condition	β-thalassaemia major, diabetes mellitus, iron overload-related cirrhosis, post-splenectomy	β-thalassaemia major, diabetes mellitus, past hepatitis C virus (HCV) infection (treated), post-splenectomy	β-thalassaemia major, diabetes mellitus, iron overload-related cirrhosis, osteoporosis, hypogonadism, post-splenectomy	N/A
Symptoms	Right upper abdominal pain, high fever with chills	Right upper abdominal pain, high-grade fever	Right upper abdominal pain, low-grade intermittent fever	N/A
Haemoglobin (g/dL)	5.5 (first presentation), 8.3 (second episode)	8.4	7.8	M: 13.8-17.2 / F: 12.1-15.1
WBC (x10³)	22 (first presentation), 31.5 (second episode)	53 (first presentation), 49.8 (second episode), 57 (third episode)	24	4.0-10.5
C-Reactive Protein (mg/dL)	32 (first presentation), 62 (second episode)	258 (first), 187 (second), 275 (third)	104	<0.5
Liver Abscess Size and Location	6 lesions (largest 2.2 × 2 cm) in Segments 4a, 5, 6, 7, 8	6.3 × 5.5 cm in Segments 6/7 (first), 6 × 4 cm in Segment 4 (second), largest 10 cm³ (third)	5 × 4.8 × 3.9 cm in Segment 2, 2.9 × 3.3 cm in Segment 3	N/A
Pathogen Identified	*Klebsiella pneumoniae* (blood and aspirate culture)	*Coliform* spp., *Pseudomonas* spp., methicillin-resistant *Staphylococcus aureus (*MRSA) (blood and aspirate culture)	*Pseudomonas* spp. (aspirate culture)	N/A
Initial Antibiotic Treatment	IV Meropenem + IV Ciprofloxacin	IV Meropenem + IV Metronidazole → IV Ceftazidime	IV Meropenem + IV Metronidazole	N/A
Recurrence	Yes (10 months later)	Yes (twice, at 8 months and later while on oral antibiotics)	No	N/A
Final Outcome	Deceased (ICU due to COVID-19 pneumonia and recurrent liver abscess)	Fully recovered after 6 weeks IV Meropenem + 3 months oral therapy	Complete resolution after IV and oral antibiotics	N/A

Case 2

A 26-year-old β-thalassaemia major male patient presented with right-sided upper abdominal pain and high-grade fever for a duration of three days. His comorbidities included diabetes mellitus with reasonable glycaemic control and a past hepatitis C infection (successfully treated in 2018). He had undergone splenectomy at the age of 19 years and was up to date with his splenectomy-related vaccinations. His haemoglobin was 8.4 g/dL, WBC was 53 × 10³, platelet count was 573 × 10³, and C-reactive protein was 258 mg/dL at the time of presentation. His coagulation profile and renal function tests were within normal ranges. Retroviral studies, melioidosis antibodies, and the Mantoux test were non-reactive. His last recorded serum ferritin, prior to the illness, was 1,200 ng/mL. A USS of the liver revealed a hypoechoic, partially liquefied focal liver lesion measuring 6.3 × 5.5 cm, overlying Segments 6/7, suggestive of a liver abscess (Figure 1). An attempt at USS-guided abscess aspiration was unsuccessful. Hence, medical management with empirical intravenous meropenem 1 g three times daily and metronidazole 500 mg three times daily was commenced. Both blood and urine cultures were negative for bacteria. CECT of the abdomen confirmed a solitary liver abscess. After completing 10 days of the initial antibiotic regimen, antibiotics were changed to intravenous ceftazidime 2 g once daily following a multidisciplinary discussion. As the patient improved symptomatically, with resolution of inflammatory markers, he was discharged on oral co-amoxicillin 625 mg TDS and ciprofloxacin 500 mg BD for another 14 days (total antibiotic duration of five weeks).

Eight months later, he again presented with a history of high-grade, intermittent, episodic fever and right-sided upper abdominal pain. On admission, WBC was 49.8 × 10³, and C-reactive protein was 187 mg/dL. Both renal and liver function tests were within normal ranges. An abdominal USS revealed a hypoechoic focal liver lesion in Segment 4, measuring 6 × 4 cm, from which 30 mL of pus was aspirated. Pus culture grew *Coliform* (species-level ID not available) and *Pseudomonas* species on the 8th and 11th days, respectively. In addition, blood cultures yielded methicillin-resistant *Staphylococcus* *aureus* (MRSA) after 29 hours of incubation. The patient was commenced on intravenous imipenem-cilastatin 1 g three times daily and intravenous ciprofloxacin 400 mg twice daily for seven days. Clinical and biochemical improvement was noted by day 10. He also developed a mild COVID-19 infection, which did not require any specific treatment during this admission. He was discharged on oral amoxicillin-clavulanic acid 625 mg TDS plus ciprofloxacin 500 mg BD for a further month.

He was re-admitted with a history of high-grade, intermittent fever and right upper abdominal pain while on the above oral antibiotic combination. This led to the detection of recurrent multiple liver abscesses on USS. The largest abscess was 10 cm³ in size. Repeated blood cultures failed to yield bacterial pathogens, but he recorded elevated inflammatory markers (WBC was 57 × 10³, and C-reactive protein was 275 mg/dL). Since *Pseudomonas* and *Coliform* were cultured in the liver aspirate fluid during the first admission, it was decided to commence intravenous meropenem 1 g TDS for six weeks via a peripherally inserted central cannula (PICC), after fulfilling MRSA eradication strategies under strict infection control measures. He responded well to meropenem, with a subsequent reduction in the size of the abscess to 5.7 × 3.4 cm. After completing six weeks of intravenous meropenem, he was discharged on oral ciprofloxacin 750 mg BD and metronidazole 400 mg TDS for three months. To date (36 months later), he has remained clinically stable and abscess-free (Table 1).

Case 3

A 46-year-old male β-thalassaemia major patient on regular monthly packed cell transfusion presented with a history of right-sided upper abdominal pain and low-grade, intermittent fever over a one-week period. He had multiple co-morbidities, including diabetes mellitus, iron overload-related cirrhosis of the liver (HBV and HCV negative), hypogonadism, and osteoporosis. He had been splenectomised at the age of 15 years and was up to date with his splenectomy-related vaccinations. His haemoglobin was 7.8 g/dL, WBC was 24 × 10³, and C-reactive protein was 104 mg/dL on admission. His renal biochemistry and coagulation profile remained within normal levels. Retroviral studies, melioidosis antibodies, and the Mantoux test were not reactive. Serum ferritin prior to the episode was 2200 ng/mL. He was empirically commenced on intravenous meropenem 1 g TDS and intravenous metronidazole 500 mg TDS. His blood and urine cultures didn’t yield pathogens after five days and 24 hours of incubation, respectively. CECT abdomen showed two lesions in Segment 2 (5 × 4.8 × 3.9 cm) and Segment 3 (2.9 × 3.3 cm), from which 15 mL of pus was aspirated. The aspirated pus sample cultured *Pseudomonas* spp. after five days of incubation at 37°C in air. After completing seven days of meropenem and metronidazole combination, based on the aspirate culture report and antibiotic sensitivity, he was switched to intravenous ceftazidime 2 g three times daily for a further 17 days. Symptomatic improvement and resolution of inflammatory markers led to his discharge to home with oral co-amoxiclav 625 mg TDS and ciprofloxacin 500 mg BD for a further 14 days. Repeat USS abdomen confirmed complete resolution of the liver abscesses (Table 1).

## Discussion

We discuss the case histories of three heavily transfused thalassaemia patients treated in our centre for liver abscesses. All three patients shared many common characteristics. All of them were young males, presented with right-sided abdominal pain and acute febrile illness associated with chills, had high ferritin suggesting iron overload, had diabetes mellitus, and had been splenectomised. Tuberculosis, melioidosis, or amoebiasis were excluded as causes for abscess formation. The abscesses were located in the right lobe of the liver (Segments 5-8). The largest liver abscess measured 10 cm³, while the smallest, recorded on a USS abdomen, was 1.7 × 2.1 cm. Although one patient had cystic duct calculus, none had a history of biliary infection or underwent surgical interventions. *Coliforms*, *Pseudomonas* species, *Klebsiella*, and MRSA were the bacterial pathogens identified in the blood or aspirate. All patients received intravenous meropenem with either metronidazole or ciprofloxacin at the onset. Later, antibiotic stepdowns were arranged based on clinical response, culture results, or antibiotic sensitivity patterns. All were sent home on an oral antibiotic course (>14 days) consisting of ciprofloxacin and co-amoxiclav to facilitate complete resolution of the underlying liver abscesses. Two out of three patients were re-admitted with possible re-emergence or recrudescence of liver abscesses, requiring further antibiotic management. One patient required PICC (peripherally inserted central catheter) line placement for antibiotic delivery, while the others were managed with peripheral venous cannulation. The delayed re-emergence or recurrence of liver abscess in the same patient was noteworthy. In two of these patients, the liver abscess re-emerged almost eight months after the first event. Neither of them had a follow-up scan in the interim period, which would have allowed us to reliably say if the new abscess was a re-emergence of the same lesion. One patient died from COVID-19 pneumonia, and in him, the liver abscess may have had a direct or indirect contribution to the death.

Two patients developing COVID-19 in the case series are coincidental, highlighting the occurrence of the pandemic during this time. Incidentally, Case 1 was the only death related to COVID-19 of a thalassaemia patient reported from Sri Lanka.

Mini-review of the literature

The review of the literature was carried out in PubMed in English, from inception through September 30, 2021, to identify case reports of thalassaemia patients with liver abscesses. References of included articles were also searched manually. Relevant articles for the topics of interest were determined by two independent reviewers (NN and HJ). PubMed tags used in queries: (MeSH) - Medical Subject Headings, (TIAB) - (("thalassaemia"(All Fields) AND "liver abscess"(All Fields)) ("1980/01/01"(PDAT) : "2021/09/30"(PDAT)). The screening of eligible publications was carried out independently by NN and MN. First, the titles and abstracts of all citations were reviewed. Next, the full text of potentially relevant citations was reviewed. Discrepancies were resolved by consensus. Data were extracted by NN and crosschecked by AP. We used a modified version of a tool for the quality appraisal of case reports [9].

We were able to collect 11 cases with liver abscesses. The age ranged from 12 to 47 years, with a male preponderance. The recorded complications in the literature included bacteraemia, pancreatitis, subphrenic abscess, pleural effusion, and meningitis. None of the patients in our case series had any of these. *Klebsiella* was the most common pathogen isolated from liver abscesses in the available literature (Table 2). In contrast, in our case series, *Klebsiella* was yielded in only one aspirate culture. Other pathogens documented in the case reports and studies included *Amoeba*, *Yersinia enterocolitica*, and *Burkholderia pseudomallei*. None of these pathogens were featured in our case series. With regard to lobar involvement within the liver, there were sparse records related to the site of involvement in most studies. Of those that did report (N = 4), most had multiple segmental involvement, with no predominance noted in either the left or right lobes of the liver. In our case series, there was predominant involvement of the right side of the liver, with Segments 5-8 affected. The data on antibiotics used for the treatment of these patients were not uniformly recorded in all studies included in the literature review. In three studies, the use of aminoglycosides, trimethoprim/sulfamethoxazole (TMP/SMZ), and third-generation cephalosporins was noted. In our case series, a newer class of carbapenems, such as meropenem, was given in combination with nitroimidazole, such as metronidazole. Most of the patients involved in the studies included in the literature review recovered following treatment and remained well on follow-up.

**Table 2:** Reported cases of thalassaemia with liver abscess during the last 30 years Year, Published year; Age, In years; M, Male; F, Female; BTM, Beta thalassaemia major; NTDT, Non transfusion dependent thalassaemia; G6PD, G6P deficiency; HbSC, HbC sickle disease; TB, Tuberculosis; MDR, Multi drug resistance; NA, Not available; TMP/SMZ, Trimethoprim/sulfamethoxazole

Year	Age	Sex	Type	Other Organs	Organisms	Antibiotic	Survival	Lobes
2010 [10]	21	M	BTM	Not mentioned	Klebsiella	Not mentioned	Live	Not mentioned
2011 [11]	16	NA	BTM	Not mentioned	Klebsiella	Not mentioned	Live	Not mentioned
2011 [11]	20	NA	BTM	Not mentioned	Klebsiella	Not mentioned	Live	Not mentioned
2011 [11]	23	NA	BTM	Not mentioned	Klebsiella	Not mentioned	Live	Not mentioned
2011 [11]	15	NA	BTM	Not mentioned	Not mentioned	Not mentioned	Live	Not mentioned
2011 [11]	19	NA	BTM	Bacteraemia	Klebsiella	Not mentioned	Live	Not mentioned
2011 [11]	22	NA	BTM	Bacteraemia	Klebsiella	Not mentioned	Live	Not mentioned
2012 [12]	25	M	BTM	Pancreas	Melioidosis	Not mentioned	Not mentioned	Not mentioned
2014 [13]	13	M	BTM	Not mentioned	Not mentioned	Not mentioned	Not mentioned	Left single
2014 [13]	15	M	BTM	Not mentioned	Not mentioned	Not mentioned	Not mentioned	Double
2013 [14]	NA	NA	BTM	Not mentioned	Klebsiella	Not mentioned	Live	Not mentioned
2004 [15]	12	M	BTM	Not mentioned	TB	MDR	Live	Multiple
2017 [16]	47	F	NTDT G6PD	Not mentioned	Klebsiella	Imipenem, Vancomycin & Metronidazole	Not mentioned	2,3,4
2020 [17]	16	M	BTM	Not mentioned	Klebsiella	Not mentioned	Live	Not mentioned
2020 [18]	17	M	HbSC	Not mentioned	Fusobacterium nucleatum	TMPSMZ, Aminoglycosides	Live	Multiple
1994 [19]	19	M	BTM	Not mentioned	Klebsiella	TMPSMZ, Aminoglycosides	Live	Not mentioned
1994 [19]	22	M	BTM	Subphrenic abscess, Pleural effusion, meningitis	Klebsiella	TMPSMZ, Aminoglycosides	Live	Not mentioned
1993 [20]	15	M	BTM	Not mentioned	Yersinia enterocolitica	3rd Gen Cephalosporine & Aminoglycoside	Live	Multiple
1998 [21]	NA	NA	BTM	Not mentioned	Amoeba	Not mentioned	Not mentioned	Not mentioned

An important observation noted in our case series was the recurrence of liver abscesses, despite prolonged, broad-spectrum antibiotic usage. All of these patients had clearly identifiable risk factors. All of the patients, in this case series, had diabetes mellitus and were splenectomised, which put them at risk of infections caused by encapsulated bacteria. There are several other possible explanations for the recurrences. Frequent venous cannulations in patients receiving regular blood transfusions can lead to fibrosis of the veins. This results in difficult peripheral venous access and often leads to multiple unsuccessful punctures, which may ultimately facilitate the entry of organisms such as *Coliform* and *E. coli*. Failure to secure a peripheral intravenous cannula is quite common among thalassaemia patients as well. This leads to loss of continuity in intravenous antibiotic therapy, especially when prolonged use is necessary - another factor that could contribute to poor healing of the abscess. The emergence of drug-resistant strains of bacteria during prolonged antibiotic courses is another factor that needs careful consideration.

There is no consensus regarding the duration of treatment with antibiotics. Short courses (two weeks) of therapy after percutaneous drainage have been successful in a small series of non-thalassaemic patients whose abscesses were completely drained [22]. However, four to six weeks of antibiotic therapy has been the recommendation for solitary lesions that have been adequately drained [23]. However, multiple abscesses, on the other hand, can be more problematic and thus require up to 12 weeks of therapy. Both the clinical and radiographic progress of the patient should guide the length of therapy. Therefore, considering all the factors, we would like to recommend that a prolonged course of intravenous antibiotics for at least six weeks is necessary for liver abscess in patients with iron-overloaded conditions such as thalassaemia, followed by another course of high-dose oral antibiotics for more than six weeks, after direct consultation with microbiology specialists.

Adequate surveillance for resolution or recurrence of the abscess is the other key factor. Adequate patient education and long-term follow-up must be arranged to ensure prevention of recurrence. It is also worth noting that smaller abscesses (<1 cm) are difficult to detect on routine USS abdomen; hence, regular follow-up and surveillance of USS abdomen must be instituted. In cases of doubt, CECT abdomen should be utilized to help identify and assess uncertain focal liver lesions.

## Conclusions

Liver abscesses are not uncommon among thalassaemia patients and can be detected early based on clinical suspicion and abdominal imaging. Successful treatment would entail isolation of the pathogen, selection of the appropriate antibiotic(s), obtaining adequate venous access for drug delivery, maintaining aseptic conditions during procedures, continuation of antibiotic therapy for an adequate duration based on clinical and radiological progress, and post-treatment vigilance to detect recurrences.

Liver abscesses in patients with thalassaemia major require prolonged courses of antibiotics, combined with careful follow-up to detect recurrence. Iron-overloaded patients with diabetes mellitus, especially those who are splenectomised, seem to be particularly vulnerable, and poor venous access makes long-term intravenous antibiotic delivery exceptionally challenging.
